# Impact of the Prepectoral Breast Reconstruction Assessment Score on Expander-Based Reconstruction Success

**DOI:** 10.3390/jcm13216466

**Published:** 2024-10-28

**Authors:** Federico Lo Torto, Gianmarco Turriziani, Sara Carella, Alessia Pagnotta, Diego Ribuffo

**Affiliations:** 1 Unit of Plastic and Reconstructive Surgery, Department of Surgery “P. Valdoni”, Policlinico Umberto I, Sapienza University of Rome, 00185 Rome, Italy; 2 Department of Plastic Surgery, USL Umbria 1, 06127 Perugia, Italy; 3 Hand and Microsurgery Unit, Jewish Hospital of Rome, 00186 Rome, Italy

**Keywords:** breast reconstruction, tissue expanders, prepectoral reconstruction, prepectoral expanders, prepectoral breast reconstruction assessment score

## Abstract

**Background/Objectives**: The rising incidence of breast cancer has led to more mastectomies and increased demand for reconstruction. While retropectoral reconstruction with expanders is common, it has complications like postoperative pain and animation deformity. Prepectoral reconstruction, aided by advancements in biological and synthetic meshes, offers a promising alternative. **Methods**: This study prospectively evaluated the “Prepectoral Breast Reconstruction Assessment Score” on 20 patients undergoing mastectomy at Policlinico Umberto I, Rome, from July 2022 to February 2024. Patients with scores between 5 and 8 were included. The procedure involved the use of ADM (Acellular Dermal Matrix) or titanium-coated polypropylene mesh, followed by postoperative expansions and final implant placement after six months. **Results**: The mean age of patients was 51.85 years, with a mean BMI of 24.145 kg/m^2^. ADM was used in 15 cases and synthetic mesh in 5. Complications were one exposure of the expander, one superficial skin necrosis and one seroma. Statistical analysis showed a trend toward fewer complications with higher scores, though this was not statistically significant (*p*-value = 0.139). **Conclusions**: Prepectoral reconstruction with expanders is a viable option, offering benefits such as reduced operating time, better volume control, and a more natural breast contour compared to the retropectoral approach. Although the trend suggests fewer complications with higher assessment scores, further studies with larger samples are needed for confirmation.

## 1. Introduction

In recent years, the increase in breast cancer incidence has been associated with an increase in the number of mastectomies [1,2].

The most common breast reconstruction is the heterologous reconstruction, using expanders and breast implants. The classic placement of these implants is the retropectoral space, below the Pectoralis Major muscle [3]. The presence of muscle between the implant and the mastectomy flaps ensures greater coverage of the implant and therefore less likelihood of its exposure in case of skin envelope necrosis. On the other hand, the retromuscular site is associated with well-known complications: postoperative pain, animation deformity (AD), implant migration, poor expansion of the lower pole, and poor definition of the inframammary fold (IMF) [4,5,6]. To overcome these problems, in the 70’s Snyderman and Gruber proposed the first prepectoral reconstructions that did not lead to the expected results, due to the high rate of complications, particularly the implant exposure [7,8]. New reconstructive perspectives opened up in 2005 with the introduction of biological and synthetic meshes. The development of these devices, together with the evolution of conservative mastectomy techniques and technological advancement, such as the use of preoperative digital mammography or indocyanine green in the evaluation of mastectomy flap stability, led to the resumption of breast reconstructions in the subcutaneous setting [9,10,11,12,13,14,15].

Although expander reconstruction remains the most used reconstructive process, a decrease in its use has been reported in favor of direct-to-implant (DTI) reconstructions in several countries [16,17]. With the long-term results of comparative studies on DTIs [16,18], the use of the expander has found its place in a new reconstructive landscape. The use of DTI, in fact, is based on a series of preoperative and intraoperative parameters that can give or not the indication for this reconstructive method [19]. Prepectoral expanders are a consequence of DTIs: this method follows the same indications as immediate prepectoral reconstruction, but it is used when a DTI cannot be performed. Various authors have described their results regarding the use of expanders in the subcutaneous space and some meta-analyses have highlighted the advantages and disadvantages of this method, but, nowadays, there are no validated protocols that point to the best type of reconstruction for each patient.

The retrospective study by Casella et al. [20] sought to formulate a risk-assessment score, based on preoperative and intraoperative parameters, in order to guide the surgeon towards the best reconstructive choice.

The aim of our study is to prospectively evaluate the effectiveness of the “Prepectoral Breast Reconstruction Assessment Score” in implant-based breast reconstruction, especially in prepectoral breast reconstructions with tissue expanders.

## 2. Materials and Methods

The study sample was obtained from the total number of patients who underwent mastectomy between July 2022 and February 2024, at the UOC of Plastic Surgery of the Policlinico Umberto I (Rome). All patients were assessed with the “Prepectoral Breast Reconstruction Assessment Score” sec. Casella, including 7 preoperative parameters and 1 intraoperative parameter. Before the mastectomy, all patients were visited by the General Surgeon and Plastic Surgeon, evaluating the individual preoperative risk factors: each parameter was assigned a score from 0 to 2. During the demolitive procedure, the surgical operators assessed the thickness of the mastectomy flaps, assigning additional points (0–2) (Table 1). The final score leads surgeons in the decision-making process, suggesting the most appropriate reconstructive procedure for that selected patient (Table 2).

Inclusion criteria: patients undergoing skin-sparing or nipple-sparing mastectomy with an overall score between 5 and 8. Exclusion criteria: patients undergoing skin-sparing or nipple-sparing mastectomy with a total score <5 or >8.

The mastectomy and the procedures on the axillary lymph nodes were performed by the General Surgeon. After the demolitive time, the validity of the mastectomy flaps was evaluated by applying the intraoperative score and patients with an overall score outside the reference range 5–8 were excluded. The Plastic Surgeon then evaluated the need to associate the expander with a biological or synthetic mesh. With the prosthetic pocket properly fitted, the expanders (Mentor, Breast Implants, Mentor Worldwide, Santa Barbara, CA, USA) wrapped by matrix were fixed to the pectoralis fascia with 3 interrupted absorbable sutures. In the presence of damaged IMF, this has been reconstructed properly by means of suturing techniques. The subcutaneous expander was always partially prefilled with Methylene Blue and Saline Solution (30–80 cc). In all cases, a suction drain was placed at the IMF level. All patients had antibiotic coverage during and after the stay: a double intravenous antibiotic therapy was performed during hospitalization (cefazolin and gentamicin), and an oral antibiotic from the day of discharge to the removal of drains. Postoperative expansions started after about 3 weeks, reaching the appropriate volume with 2–4 outpatient sessions. After about 6 months, the second surgical time for replacing the expander with the definitive prosthesis was scheduled, possibly associating a contralateral mammoplasty for adjustment. In the presence of particularly superficial implants or after radiotherapy, 1 or more procedures of autologous adipose tissue grafting were performed before the placement of the permanent implant. Follow-up visits were performed at 1, 3, 6, and 12 months.

In the postoperative time, the main complications were evaluated: skin necrosis, infection, hematoma, and seroma (>30 mL after 2 weeks from surgery).

A statistical analysis was performed to evaluate the association between preoperative/intraoperative score and postoperative complications using Binary Logistic Regression. The SPSS software (Version 29.0) was used to perform the analysis: in order to statistically validate the score, significant results of *p*-value < 0.05 were considered.

## 3. Results

A total of 20 patients were found to be suitable for two-stage reconstruction with prepectoral expanders, achieving a score between 5 and 8. Mean age was 51.85 years (range 35–71): 7 patients with age < 50 years (35%), 12 patients aged 50–70 years (60%), and 1 patient was > 70 years (5%). The mean BMI was 24.145 kg/m^2^ (range 19–33.2): 6 patients < 22 kg/m^2^ (30%), 7 patients > 25 kg/m^2^ (35%), and 7 patients 22–25 kg/m^2^ (35%). The mean mastectomy flap thickness was 1.09 cm: 10 patients had mastectomy flaps < 1 cm, 10 patients had between 1 and 2 cm, and none had > 2 cm. Seven patients (35%) had Type II diabetes mellitus, four patients (20%) had active smoking habits while ten patients (50%) were ex-smokers. Three patients (15%) had breast ptosis, eight (40%) had a previous breast surgery (4 wide local excisions, 3 contralateral mastectomies, 2 QUART, 1 breast augmentation), five had radiotherapy. Patient characteristics are summarized in Table 3.

The average hospitalization stay was 2.7 days, and, in all cases, the double intravenous antibiotic therapy was performed for 2–3 days of hospitalization. ADMs were used in 15 cases and titanium-coated polypropylene meshes in 5 cases. Drains were removed after an average of 9 days. Postoperative pain was treated with classic painkillers, without the use of opioids.

**Figure 1:**
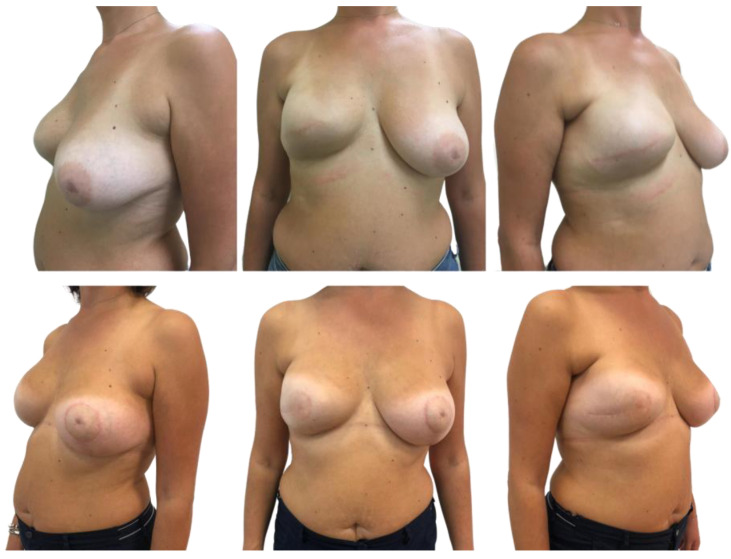
Right prepectoral expander: I and II reconstructive time.

**Figure 2:**
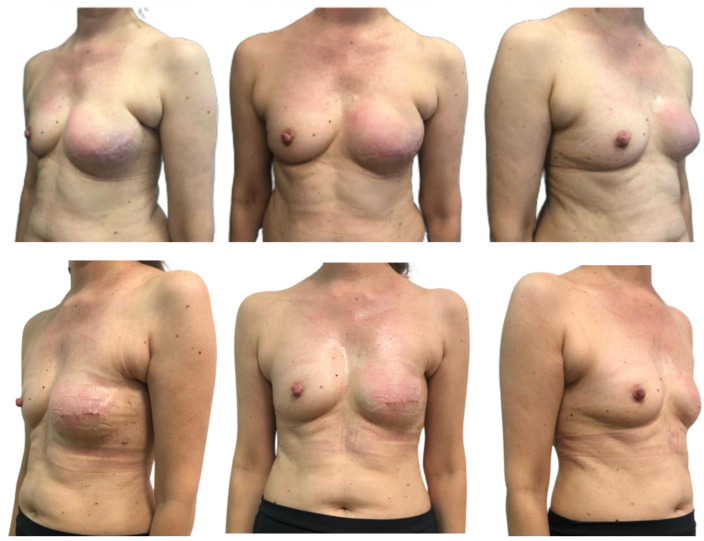
Left prepectoral expander: I and II reconstructive time.

No infections or hematomas occurred. There was superficial skin necrosis in one patient who was treated conservatively. Full-thickness skin necrosis of the mastectomy flap led to exposure of the expander in one case, and the implant was subsequently removed. Seroma occurred in one patient with synthetic mesh. Final follow-up was at least 6 months (Figure 1 and Figure 2).

Binary Logistic Regression evaluated the association between “total score” and “complications” to determine whether the Prepectoral Breast Reconstruction Assessment Score is a significant predictor of complications. The coefficient of the total score was −1.1620, while the *p*-value was 0.139.

## 4. Discussion

The patients who were found to be eligible for the study scored between 5 and 8 on the Prepectoral Breast Reconstruction Assessment Score. This score was introduced in the 2020 study of Casella et al. [20] and carried out on 352 patients, but in that case, it was applied retrospectively to compare scores obtained with previously performed reconstructive surgeries. This tool is the first step towards an objective evaluation of reconstructive possibilities and can help surgeons to choose the most appropriate technique. By stratifying patients into risk classes, the score gives us clearer indications on the pre- and intraoperative factors to be evaluated in order to obtain an optimal prepectoral reconstruction. Older patients with a high BMI, active smokers or those with diabetes mellitus usually have lower quality tissues due to the impairment of microcirculation, with higher risk of complications, such as necrosis of the mastectomy flap, infection, and implant exposure. Obesity (BMI > 35 kg/m^2^) is considered an exclusion criterion for prepectoral reconstruction and each point of increase in the BMI corresponds to a 5.9% increase in the risk of complications and a 7.9% increase in the risk of failure of the procedure. Similarly, even a reduced BMI < 22 kg/m^2^ is associated with a high risk of developing surgical complications [21,22,23]. The assessment score also considers the degree of breast ptosis that would require a skin-reducing mastectomy (>II) and is associated with a higher risk of exposure of the implant at the level of the IMF [24,25]. Diabetes mellitus, especially when poorly controlled with high blood glucose levels documented by HbA1c values > 7%, affects wound healing and blood supply to the skin envelope. Active smoking is a known contraindication to prepectoral reconstruction. Considering the patient’s medical history, those who have undergone previous breast surgery or preoperative radiation therapy receive 0 points in this score because they have a higher risk of wound dehiscence and infection [26,27]. Postoperative radiotherapy is not included in the criteria of this score because it is considered a necessary treatment after mastectomy. Recent studies [28,29,30,31,32] show how the preoperative RT should not be considered an absolute contraindication for prepectoral reconstruction; even better, this surgical technique can be a better option than the subpectoral reconstruction: in fact, the irradiated muscle can become fibrous and apply force on the implant, causing a malposition and a higher risk of capsular contracture [33]. The thickness of the mastectomy flap and its viability are essential for an optimal reconstruction [34,35,36,37,38]; a good flap thickness and enough subcutaneous adipose tissue are fundamental factors for prepectoral reconstruction. Zero points are awarded if the flap is <1 cm and/or if the viability of the tissue is considered poor due to reduced bleeding of the margins, 1 point if the thickness is 1–2 cm, 2 points if >2 cm. From the experience of this preliminary study, we have confirmed that prepectoral reconstruction has considerable advantages [39,40,41,42,43]: the surgical procedure is less invasive, has easier, shorter operating times, and no dissection of the pectoral muscle resulting in less bleeding, less pain, and faster postoperative recovery. Since the contraction of the muscle does not involve the implant, the risk of migration of the same upwards is reduced and, crucially, the incidence of animation deformity is significantly reduced [6,44]. The pectoralis muscle is left in its natural location and the implant replaces the natural position of the mammary gland: this ensures greater comfort, better breast projection and a better aesthetic outcome, giving a more natural look to the breast, as already highlighted in other publications [45,46]. No clinically significant capsular contractures were detected at the final follow up: this may be related to the fact that the membrane wrapping the implant integrates with the host tissues, preventing pericapsular fibrosis [47,48]. In some cases, when the expander was replaced with the permanent implant, there was no need to perform surgery on the contralateral breast, since the residual asymmetry for the patient was already acceptable: in fact, the more natural ptosis of the implant allows surgeons to achieve a certain degree of symmetry and, consequently, “save” an additional procedure.

Compared to the data in the literature, the following results can also be highlighted. There was a lower rate of necrosis of the mastectomy flaps and nipple compared to other surgical procedures such as autologous reconstructions [49,50]. The expander allows better volume control both intraoperatively and postoperatively, requiring fewer expansion sessions [51,52]. Patients with a prepectoral expander require fewer postoperative controls and have a shorter time interval between the I and II reconstructive time [53,54]. The breast expander can initially be used to prepare the site for subsequent autologous reconstruction, for example with a DIEP flap. This helps to reduce risks and improve the overall outcome of autologous reconstruction and provides greater flexibility in treatment path planning [55,56,57,58,59].

The statistical analysis shows that the negative coefficient (−1.1620) suggests that as the total score increases, the likelihood of developing complications decreases, but this result is not statistically significant. The *p*-value of 0.139 indicates that the association between total score and complications is not statistically significant at 5% significance level. However, there is a trend that may be relevant with a larger sample.

## 5. Conclusions

Although there has been an increase in DTI reconstructions, two-step heterologous reconstruction with expanders is confirmed as an important and versatile reconstructive resource in cases where DTI is not indicated. In this scenario, the breast expander acquires a new importance by finding use in a wider range of situations than its classic use. Compared to the traditional retropectoral approach, the functional advantages of this technique are the flexibility and better volume control, reduction of operating time, less pain, less postoperative controls, reduction of the risk of migration of the implant and animation deformity, possibility to associate the expander with autologous reconstruction techniques in multiple steps and better post-radiotherapy results in combination with lipofilling. In addition to the functional aspect, this reconstructive technique also gives a more natural breast contour and a better aesthetic result. The implant of the device above the muscle, in fact, reproduces the anatomical position of the mammary gland providing a more comfortable and harmonious result. The results show a trend of complications decreasing with increasing score: further studies with larger samples are needed to statistically confirm this trend.

## Figures and Tables

**Table 1:** Assessment of individual preoperative and intraoperative risk factors for implant-based prepectoral breast reconstruction failure.

Risk Factor	Score			Range of Score per Factor
	0	1	2	
Patient’s age, yr	>70	50–70	<50	0–2
Diabetes	Yes	No		0–1
Smoker	Current smoker	Ex-smoker	Never smoker	0–2
BMI	Low: <22	High: >25	Medium: 22–25	0–2
Breast ptosis: indication for skin-reducing mastectomy	Yes	No		0–1
Previous breast surgery	Yes	No		0–1
Radiotherapy	Yes	No		0–1
Mastectomy flap thickness, cm	<1	1–2	>2	0–2

BMI, body mass index.

**Table 2:** Individual patient’s score for selection of the safest implant-based breast reconstruction.

Score	Implant-Based Breast Reconstruction
0–4	No indication for prepectoral reconstruction; submuscular placement of the implant
5–8	Two-stage reconstruction with prepectoral tissue expander first and subcutaneous definitive prosthesis second
9–12	Prepectoral direct-to-implant breast reconstruction

**Table 3:** Patient characteristics.

Patient	Age	Diabetes	Smoke	BMI (kg/m^2^)	Breast Ptosis	Previous Breast Surgeries	Radiotherapy	Mastectomy Flap Thickness (cm)	Complications	Total Score
1	35	no	yes	27.2	no	yes	no	1.30		7
2	48	no	yes	23	yes	no	no	1.2		8
3	55	yes	no	26.1	no	yes	yes	1.4		6
4	57	no	ex	27.3	no	no	no	0.9		7
5	46	yes	ex	24.6	yes	no	yes	0.8	Superficial skin necrosis	6
6	63	yes	yes	25.7	no	no	no	0.9	Breast expander exposure and removal	5
7	42	no	ex	22.3	no	no	yes	0.9		8
8	45	no	ex	21.8	no	yes	no	1.3		7
9	38	no	no	21.7	no	no	yes	0.8		7
10	52	no	ex	26.3	no	no	no	1.7		8
11	56	yes	no	28.5	no	no	no	1.3		8
12	55	no	no	21	no	yes	no	1.2		7
13	58	yes	ex	24.3	no	no	no	0.8	Seroma	7
14	49	no	no	19	no	yes	yes	0.8		6
15	52	no	ex	23.2	no	no	no	0.7		8
16	51	no	no	19.7	no	yes	no	0.8		6
17	53	no	ex	22	no	yes	no	0.9		7
18	61	yes	ex	24.1	no	yes	no	1.4		7
19	71	no	yes	21.9	no	no	no	1.1		5
20	50	yes	ex	33.2	yes	no	no	1.6		6

## Data Availability

Data are unavailable due to privacy restrictions.
